# Ferroelectric domain states of a tetragonal BiFeO_3_ thin film investigated by second harmonic generation microscopy

**DOI:** 10.1186/s11671-017-2126-5

**Published:** 2017-05-15

**Authors:** Chang Jae Roh, Sun Young Hamh, Chang-Soo Woo, Kwang-Eun Kim, Chan-Ho Yang, Jong Seok Lee

**Affiliations:** 1 Department of Physics and Photon Science, Gwangju Institute of Science and Technology (GIST), 123 Cheomdangwagi-ro, Buk-gu, Gwangju 61005 Korea; 2 Department of Physics, Korea Advanced Institute of Science and Technology (KAIST), 291 Daehak-ro, Yuseong-gu, Daejeon 305-701 Korea

**Keywords:** Ferroelectrics, BiFeO_3_, Thin film, Second harmonic generation, Multi-domain, 77.84.-s, 77.55.Nv, 78.20.-e

## Abstract

We investigate the ferroelectric state of a tetragonal BiFeO_3_ thin film grown on a LaAlO_3_ (001) substrate using an optical second harmonic generation (SHG) microscope. Whereas the ferroelectric state of this material hosts nanometer-sized domains which again form micrometer-sized domains of four different configurations, we could figure out the characteristic features of each domain from the SHG mapping with various sizes of the probe beam, i.e., from 0.7 to 3.9 μm in its diameter. In particular, we demonstrate that a single micrometer-sized domain contributes to the SHG as a coherent summation of the constituent nanometer-sized domains, and multi-micrometer-sized domains contribute to the SHG as an incoherent summation of each micro-domain.

## Background

Ferroelectrics have a great importance in both fundamental research and technical applications. Among them, BiFeO_3_ (BFO) has attracted a large attention since it hosts, as a multiferroic material, both ferroelectric and antiferromagnetic properties at the same time; it undergoes a ferroelectric phase transition at 1103 K and antiferromagnetic phase transition at 643 K [1–3]. In particular, it exhibits a large magnetoelectric effect even at room temperature [3–5] and can be used for novel functional devices, for example, in a multi-storage information technology. As in other ferroelectric materials, BFO is known to have several types of ferroelectric domains with a size ranging from nanometer to micrometer scale [6, 7]. Such a local ferroelectric domain distribution and the characteristics of individual domains have been characterized often by using piezoresponse force microscopy (PFM) with controls of temperature, electric field, and crystal strain, which revealed several intriguing phenomena, such as conducting domain wall, flexoelectric effect, and morphotropic phase boundaries [8–10].

As an alternative technique to investigate the ferroelectric properties, an optical second harmonic generation (SHG) also has been widely adopted [11–14]. The ferroelectric state with no inversion symmetry usually provides strong second harmonic signals which show specific anisotropic patterns depending on the symmetry of the material. As a non-contact optical method, this technique can be usefully exploited in the characterization of ferroelectric or polar systems with a large leakage current. Since the SHG process is allowed only when the spatial inversion symmetry is broken, it has been used also to examine the symmetry lowering at the (sub)nanometer-scale surface or interfacial state of centrosymmetric bulk or thin films [15]. Furthermore, the nanometer-scale domain distribution has been successfully demonstrated by using the SHG technique combined with a scanning probe microscope [16]. Nevertheless, it is usually difficult to investigate the individual domains by using the conventional SHG microscope as the spatial resolution is limited by a fundamental diffraction which is about several hundred nanometers in the visible spectral range [17]. SHG studies on BiFeO_3_ have been performed by several research groups who could provide detailed symmetry information of tetragonal-like and rhombohedral-like phases, but it should be noted that most of the works have been done with assumptions of the homogeneous and coherent contributions of constituent domains [18, 19].

In this paper, we demonstrate that the distribution and characteristics of ferroelectric domains for the tetragonal-like BiFeO_3_ (T-BFO) can be investigated by using a conventional SHG microscope with proper adjustments of probe beam sizes with respect to the domain size. Since this T-BFO hosts nanometer-sized domains which form specific patterns of micrometer-sized domains, it provides an excellent environment to address how the SHG responses are contributed to by each ferroelectric domain of different sizes and their mixtures. We mapped the sample with a probe beam of several sizes and found large position-dependent variations of the SHG signal which originate from distinct characteristics of the domain distribution. By considering the coherent and incoherent contributions of each domain to the SHG response, we could successfully explain such experimental results. We therefore expect that symmetry information of the individual nanometer-sized domain can be obtained even from the far-field microscopic measurement provided that the proper modeling can be chosen and applied between the incoherent and the coherent approaches.

## Methods

**Fig. 1:**
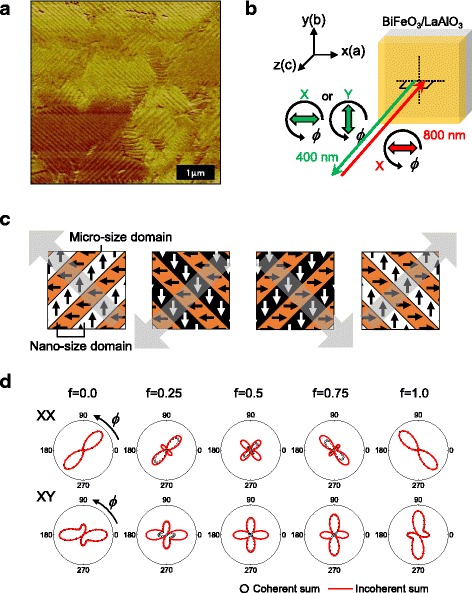
Ferroelectric domain distribution in a tetragonal BiFeO_3_ film and expected second harmonic generation results as coherent and incoherent contributions of multi-domains. **a** Piezoelectric force microscope image. *Stripe-patterned* domains are seen with two distinct orientations. **b** Schematic description of the second harmonic generation experiment. **c** Four types of *stripe patterns* of ferroelectric domains. **d** Polar plots of simulated second harmonic responses depending on a portion of different domains. Both coherent and incoherent summations are presented

The T-BiFeO_3_ thin film is grown on a LaAlO_3_ (001) substrate by using a pulsed laser deposition technique of which details can be found elsewhere [4]. The thickness of T-BFO film is about 30 nm. For the SHG experiment, fundamental light of 800 nm wavelength illuminates the sample in a normal incidence, and second harmonic light is detected in reflection geometry. We used laser pulses from the Ti:sapphire laser system for fundamental light which has a pulse width of about 30 fs and a repetition rate of 80 MHz. It has a power of about 20 mW and is focused down to the diffraction limit using an objective lens with a magnification of ×50 and a 0.75 numerical aperture. Polarization states of fundamental and second harmonic light are set to be parallel (XX) or orthogonal (XY) as shown in Fig. 1a.

## Results and discussion

We first monitored a distribution of the ferroelectric domains by using a scanning probe microscope (Bruker, MultiMode-V). PFM measurements were performed at a scan rate of 3 μm/s using Pt-coated Si conductive tips (MikroMasch, NSC35) with applying an ac driving voltage of 3 V_pp_ at a frequency of 12 kHz. Figure 1a displays the PFM image of T-BFO obtained at room temperature. It shows that ferroelectric domains form stripe patterns in a submicron length scale and the larger micron-scale areas are distributed with typical stripe orientations along 45° or 135°. In Fig. 1c, we schematically draw four types of such domain patterns. Owing to the monoclinic distortion, T-BFO has an in-plane component of the polarization as well as the polarization along the *c*-axis [7]. As the normal incidence in SHG measurement, a configuration adopted in this work is not sensitive to the out-of-plane polarization component; we here restrict our interest only in the in-plane component. We consider first a nanometer-sized domain with a polarization along the *a*- or *b*-axis, and their head-to-tail combination with a domain wall formed along the diagonal directions. Consistently with the PFM result, we hence consider four possible combinations of such domains as depicted in Fig. 1c. It should be noted that each stripe pattern is distributed over a few micrometers or even tens of micrometer scales as shown in Fig. 1a.

Before showing the experimental result, let us discuss the SHG response simulations of T-BFO (C_1h_ point group) with a consideration of each domain configuration [20]. Intensity of second harmonic (SH) light *I*(2ω) is given in proportion to the induced SH polarization *P*(2ω) as *I*(2ω) ∝ |*P*(2ω)|^2^ = |*χ*_*ijk*_ *E*_*j*_ *E*_*k*_|^2^, where *χ*_*ijk*_ represents the nonlinear susceptibility of the material, *E*_*i*_ is the electric field component of the fundamental light, and *i*, *j*, and *k* denote the crystallographic axes. The transverse coherence length *L*_*T*_ is determined as *L*_*T*_ = *λR*/2*D* [21]. Here, *D* is a size of the domain which acts as an SH light source and *λ* is a wavelength of SH light, i.e., 400 nm. *R* denotes a distance (0.38 mm) from the sample surface to the objective lens. Note that the coherence length is comparable to the objective lens size (625 μm in its diameter) when *D* is about 100 nm. Therefore, when we consider the contributions of multi-domains to the SHG, it is reasonable to assume that each nanometer-sized domain having a size of about 50 nm contributes to the SHG responses coherently as *I*(2ω) ∝ (*P*_1_ + *P*_2_)^2^, whereas micro-domains contribute to *I*(2ω) incoherently as *I*(2ω) = *P*_1_^2^ + *P*_2_^2^. Here, *P*_1_ and *P*_2_ imply the induced SH polarizations of each nano- or micro-domain when only two types of domains are considered.

Figure 1d shows the azimuth-dependent *I*(2ω) obtained by considering such coherent and incoherent contributions of different domains. For the leftmost case, we consider the coherent summation of two nanometer-sized domains corresponding to the first configuration listed in Fig. 1c. The polarization direction of each nanometer-sized single domain is along the *x*- and *y*-axes. We take *χ*_xxx_ = 0.35, *χ*_xyy_ = 1.0, and *χ*_*yxy*_ = 0.4 for the domain with the *x*-axis polarization and *χ*_yyy_ = −0.35, *χ*_yxx_ = −1.0, and *χ*_xyx_ = −0.4 for the domain with the *y*-axis polarization. All other susceptibility components are assumed to be zero. For the rightmost case, we consider the fourth configuration of Fig. 1c with the same values of *χ*_xxx_, *χ*_xyy_, and *χ*_yxy_ with changes in the sign of *χ*_yyy_, *χ*_yxx_, and *χ*_xyx_. The azimuth-dependent *I*(2ω) for the former and latter cases are displayed in the first and fifth plots in Fig. 1d; the XX response in each configuration has maximum values along the direction perpendicular to the net polarization axis because |*χ*_xxx_| < |*χ*_xyy_| and |*χ*_yyy_| < |*χ*_yxx_|. With these two cases as end members, we consider coherent and incoherent summations with different portions *f* (0.0 < *f* < 1.0) or 1-*f* of the contribution from each end member. In the intermediate cases with *f* = 0.75, 0.5, and 0.25, the incoherent summation leads to the finite value of the minimum *I*(2ω) and more swollen lobe shape compared to the coherent summation.

**Fig. 2:**
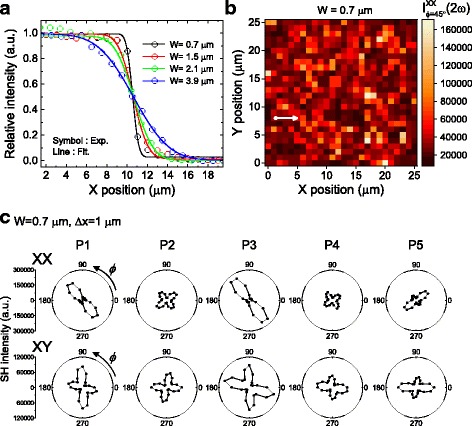
Second harmonic generation (SHG) results obtained with a beam size of 0.7 μm. **a** Knife-edge experimental results to characterize the beam size. **b** The SHG intensity mapping image obtained with a beam size 0.7 μm over the area of 25 × 25 μm^2^. **c** Azimuth-dependent polar plots of the SHG intensity obtained at different sample positions along the *arrow* in **b**

From now on, we present the experimental SHG results and discuss them based on the coherent and incoherent analyses of the multi-domain contribution. Before each SHG measurement, we characterized the beam size of fundamental light at the sample position using a knife-edge method [22]. Figure 2a shows an intensity (open symbols) obtained with a displacement of the knife edge by 1 μm. We fit the results assuming the Gaussian distribution of the beam intensity and estimate the beam size *W*, a width defined by two points having the 1/*e* ^2^ intensity of the maximum value as 0.7, 1.5, 2.1, and 3.9 μm.

Figure 2b displays a two-dimensional distribution of *I*(2ω) obtained with *W* = 0.7 μm. The displacement for each step is 1 μm, and the mapping area is 25 × 25 μm^2^. The sample azimuth Ф is fixed as 45° in the XX geometry. *I*(2ω) exhibits a large variation with no discernible pattern. These results clearly indicate that the domain size in the probed area is comparable to the beam size.

We can get a deeper insight into the ferroelectric domain by examining the azimuth-dependent of *I*(2ω) at each sample position. Figure 2c displays the results obtained at five representative positions. Although each point is displaced by 1 μm from neighboring points, the SHG results vary quite drastically from a point to a point. Looking at the results of the XX geometry, although *P*_1_, *P*_3_, and *P*_5_ exhibit twofold symmetry, *P*_2_ and *P*_4_ show a fourfold symmetry. The behaviors in the XY geometry are relatively less distinct. Nevertheless, *P*_1_ and *P*_3_ have a main lobe at Ф = 135° (315°); *P*_5_ has the corresponding maximum at 45° (225°). These results strongly suggest that each measured point has distinct preferred planar orientations of the ferroelectric polarization. Actually, the patterns of the experimental result can be identified by the simulation results shown in Fig. 1d; *P*_1_ (*P*_3_) and *P*_5_ correspond to the case *f* = 1.0 and 0.0, respectively. Also, *P*_2_ and *P*_4_ can be identified as the case *f* = 0.5 (incoherent). In other words, the results of *P*_1_ and *P*_5_ pick up the single configuration of four possible micro-domains shown in Fig. 1c, and the measurement at *P*_2_ and *P*_4_ covers two (or more) micro-domains. Considering the coherence length, it is expected that such micro-domains contribute to the SHG response incoherently. From these results, we therefore confirm that nano-domains compose a single micro-domain and contribute to the SH response coherently, whereas micro-domains contribute to the SHG response incoherently. Furthermore, the part of symmetry information of a single nano-sized domain can be obtained from the analysis of the single micro-domain contribution; the simulation results in Fig. 1 give that *χ*_xxx_ = 0.35, *χ*_xyy_ = 1.0, and *χ*_yxy_ = 0.4 for the nano-domain with the *x*-axis polarization. (In the XY geometry, the relative intensity at Ф = 45° and 135° appears oppositely to the simulation results in Fig. 1d. This discrepancy requires further analysis.)

**Fig. 3:**
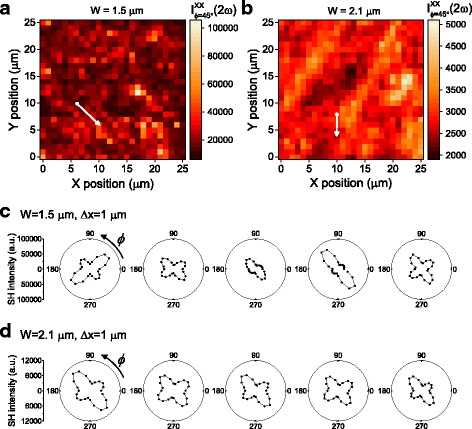
Second harmonic generation (SHG) results obtained with beam sizes of 1.5 and 2.1 μm. **a**, **b** The SHG intensity mapping image obtained with beam sizes of 1.5 and 2.1 μm, respectively. **c**, **d** Azimuth-dependent polar plots of the SHG intensity obtained at different sample positions with beam sizes of 1.5 and 2.1 μm, respectively, along the *arrows* in each figure

We increased the beam size and monitored how the SHG response varies depending on it. Figure 3a, b show the results of the two-dimensional mapping obtained with *W* = 1.5 and 2.1 μm, respectively. Here, the interval between two neighboring points is kept as the same, i.e., Δ*x* = 1 μm, and the power of fundamental light is also maintained. As the beam size increases from *W* = 0.7 to 1.5 and to 2.1 μm, *I*(2ω) decreases as expected from the smaller beam fluence. Also, the distribution of *I*(2ω) becomes to have a less position dependence.

Azimuth-dependent SHG patterns also show systematic variations as the beam size increases. For *W* = 1.5 μm, we can still observe the strong position dependence; *P*_1_ and *P*_4_ reflect two distinct micro-domains, and the other points can be considered as mixtures of such micro-domains. Compared with the results for *W* = 0.7 μm, it is clear that the incoherent contributions become more discernible for *W* = 1.5 μm; the minimum value of the SHG intensity becomes non-zero, and it is much larger than for *W* = 0.7 μm. For *W* = 2.1 μm, such tendency becomes more pronounced; there is almost no position dependence as shown in Fig. 3d. This is probably due to the influence of the dominance of a single micro-domain over the entire probing area. With *W* = 0.7 μm, a single micro-domain can be probed, and hence, the SHG intensity is determined by the coherent contribution from the constituent nano-sized domains. With *W* = 1.5 μm and 2.1 μm, on the other hand, several micro-domains are probed together, and they contribute to the SHG intensity as an incoherent summation.

**Fig. 4:**
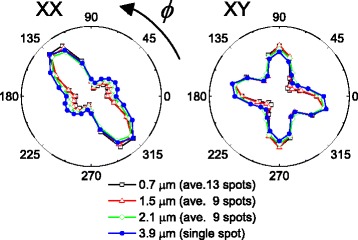
Second harmonic generation (SHG) results obtained with a beam size of 3.9 μm. Also shown are the three other curves for the different beam sizes which are obtained as averages over several points

As a final test, we further increased the probe beam size up to *W* = 3.9 μm of which result is displayed as closed circles in Fig. 4. Also shown are the results for smaller *W*s which are averaged over several points around the beam central position for *W* = 3.9 μm. Interestingly, such averaged results for *W* = 0.7, 1.5, and 2.1 μm appear quite similarly in both XX and XY geometry with the single measurement results for *W* = 3.9 μm. As this average process of the measured SH intensity is identical to the incoherent summation of each micro-domain contribution, this agreement confirms our understanding of the incoherent contribution to the SHG responses of multi-micro-domains.

## Conclusions

We investigated the ferroelectric domains of a tetragonal BiFeO_3_ film by using a second harmonic generation (SHG) technique. Whereas the normal incidence SHG measurement provides us with clear information about the in-plane component of the ferroelectric polarization, we found large variations of the SHG responses from a point to a point of the sample. This clearly indicates the inhomogeneity of the domain distribution. By reducing the beam size down to 0.7 μm, we demonstrated that the observed SHG results could reveal the symmetry characteristic of individual micrometer-sized domains which is determined by types of constituent nanometer-sized domains. By increasing the beam size up to 3.9 μm, we found the SHG response with a much less position dependence. Actually, we could reproduce this SHG result obtained with a 3.9 μm beam size with averages of several results obtained with smaller beam sizes and confirmed that each micro-domain contributes to the SHG responses incoherently. Therefore, we can have a chance to retrieve symmetry information of the individual nanometer-sized or even micrometer-sized domain if we can apply a proper model between coherent and incoherent analysis of the SHG results.

## Abbreviations

BFO: BiFeO3
PFM: Piezoresponse force microscopy
SH: Second harmonic
SHG: Second harmonic generation
T-BFO: Tetragonal BiFeO_3_
XX: Parallel
XY: Orthogonal
